# A novel surgical technique to resolve mucosal fenestration of a root apex: Apical tunnel surgery: A case report

**DOI:** 10.1097/MD.0000000000039073

**Published:** 2024-07-26

**Authors:** Jie Chen, Gaoying Ran, Jingxin Weng, Xiaohao Liu, Chengjie Xie

**Affiliations:** a Department of Periodontics, Stomatological Hospital, School of Stomatology, Southern Medical University, Guangzhou, P. R. China.

**Keywords:** microsurgery, minimally invasive surgery, Mucosal fenestration, root apex exposure, tunnel technique

## Abstract

**Rationale:**

Endodontic surgery, which includes apex resection, retro-fill and some regeneration procedures, is a traditional way to deal with apex fenestration. The endodontic surgery could bring large flap, curtate root length, non-healing mucosa and soft tissue deficiency in the apex area. Other treatment options might be considered according to different etiological factors. Mucogingival surgery provides some ideas in accumulation of soft and hard tissues, especially some unique methods such as “tunnel technique” bringing us a view of minimal invasive surgery approach. A novel surgery named “apical tunnel surgery” was reported here to resolve a root apex exposure with the tunnel-like technique.

**Patient concerns:**

A young female complained about root exposure of upper right anterior tooth without history of trauma or orthodontic treatment.

**Diagnosis:**

The intraoral examination revealed a buccal root apex exposure about 3mm in diameter of #12 (FDI teeth numbering system). The tooth was slightly dark with Class 1 mobility. The periodontal situation was good and the occlusion check revealed no traumatic bite on #12. The cone-beam computed tomography (CBCT) showed a bone fenestration from the buccally lower 1/2 root surface to the apex and bone absorption around the apex. It also revealed a bone contour deficiency in #12 area.

**Interventions:**

Root canal treatment, root surface debridement, and soft tissue combined with hard tissue accumulation were carried out in one tunnel-like surgery.

**Outcomes:**

Examination of 12-month follow-up showed a healed and thickened mucosa in the buccally apical region and CBCT showed the continuous lamina dura occupied the buccal aspect of #12 root apex.

**Lessons:**

This new apical tunnel surgery provided soft and hard tissue accumulation in one minimal invasive way in the apex exposure case caused by bone fenestration and thin mucosa.

## 1. Introduction

Apical fenestration is described as the root apex of the relevant tooth exposed by defects of the alveolar bone. Apical fenestrations are commonly found in the buccal aspect of the maxillary teeth.^[1]^ When mucosal fenestration is concomitant with apical fenestration, the situation would be more complex because the root apex is exposed in the oral environment. An apical mucosal fenestration may arise from physiological or pathological factors, such as pulp lesions, orthodontic procedures, periodontitis, tooth trauma, thin alveolar bone, buccally tilted root, occlusal trauma or laser therapy during depigmentation and gingivoplasty procedures.^[2,3]^ Apical fenestrations are most commonly caused by pulp lesions and associated with non-healing sinus tracts with symptoms as tenderness, occlusal pain and soft tissue defects.^[4]^ The management usually consists of a normative root canal treatment (RCT), root-end resection, retrofill, exposed root surface debridement, guided tissue regeneration (GTR) and mucosal grafting procedure.^[5–8]^ these managements are often required with large open flap and special surgical instruments, and bring a result of mucosa scars and shortened root length. Cases caused by other factors remain unclear whether apicoectomy and retrofill are necessary in the management procedure, especially those cases with physiological reasons. Mucogingival surgery provides some ideas in accumulation of soft and hard tissues to modify the physiological deficiency, such as “tunnel technique” bringing us a view of minimal invasive surgical option.^[9]^ We reported an “apical tunnel surgery” to resolve an apex exposure in one minimal invasive surgery, and the surgical details including flap design and choice of grafts were discussed.

## 2. Case report

**Figure 1.**
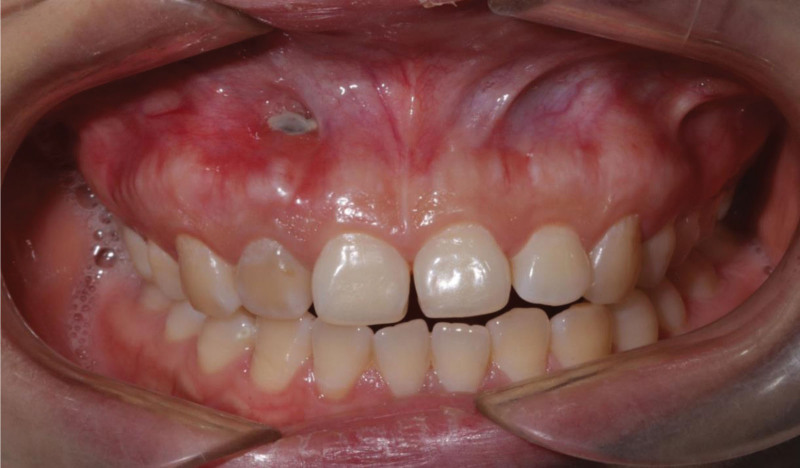
The intraoral photograph at the first visit: an apex fenestration on the buccally apical region of #12 with a dark color.

**Figure 2.**
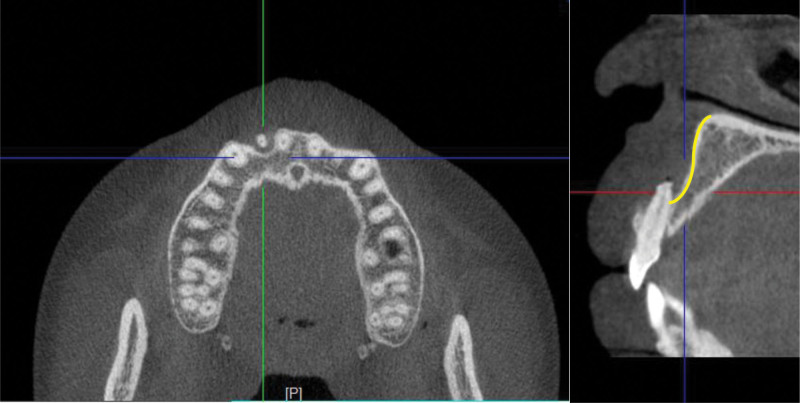
The CBCT at the first visit: bone defect around the apex and the bone fenestration on the buccally lower 1/2 root surface. The bone contour deficiency was indicated with a yellow line. CBCT = cone-beam computed tomography.

The presented patient was a 26-year-old Chinese female, who complained about slight tenderness and impaired aesthetics of right maxillary anterior tooth. The intraoral examination revealed Class 1 mobility and a 3 mm × 5 mm apical fenestration of #12 (FDI teeth numbering system), with a slightly darker color than #22 (Fig. 1). #12 was found to be periodontally sound with a probing depth of 1–2 mm and no bleeding on probing. The occlusion check revealed no early contact or traumatic bite on #12, but an open bite on #21-#23. Cone-beam computed tomography (CBCT) showed there was a bone fenestration on the buccally lower 1/2 root surface and a bone defect in the apical palatal region (Fig. 2). The patient firmly denied a history of past traumatic injuries and orthodontic treatment. According to the patient wish, combined periodonto-endodontic therapy was chosen.

**Figure 3.**
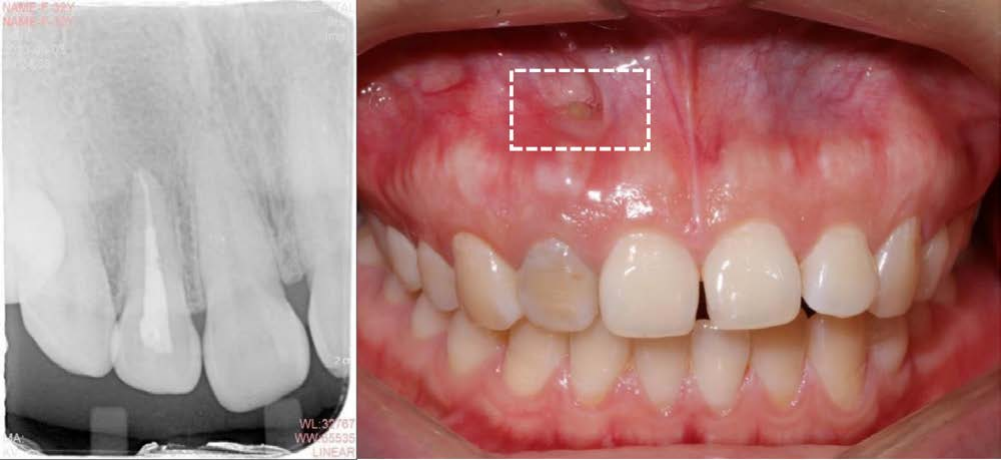
The periapical radiograph after root canal treatment and the pre-surgical intraoral photograph after calculus removed. The range of the tunnel pouch was indicated with a white dot rectangle.

**Figure 4.**
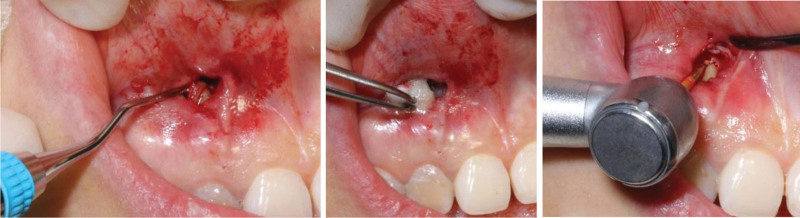
Debridement of the root apex with curettes and EDTA treating, and the medullary foramina were prepared. EDTA = ethylenediamine tetraacetic acid.

**Figure 5.**
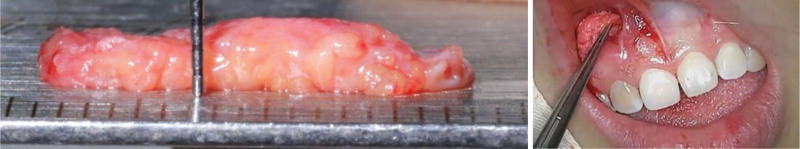
A 15 mm × 8 mm × 2.5 mm CTG with periosteum was harvested and pulled into the mesial side of the tunnel pouch. CTG = connective tissue graft.

**Figure 6.**
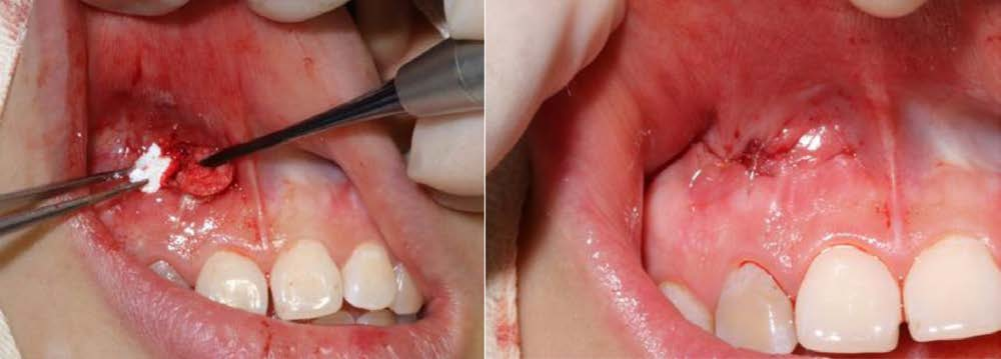
The collagen bone graft was putting into the bone defect and the CTG was pulled into the distal side of the pouch with the final closure sutures. CTG = connective tissue graft.

**Figure 7.**
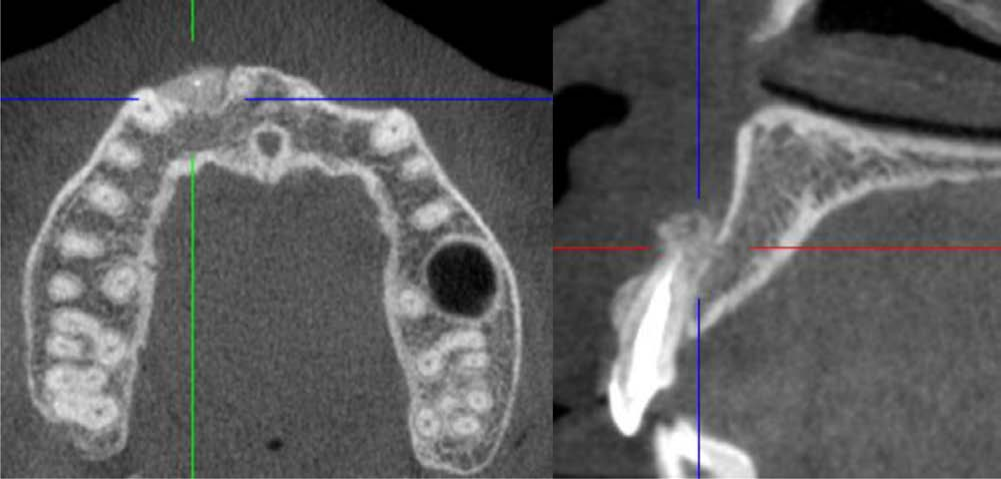
CBCT reconstruction immediately after operation demonstrated the high-density particulate matter surrounding the apex region. CBCT = cone-beam computed tomography.

**Figure 8.**
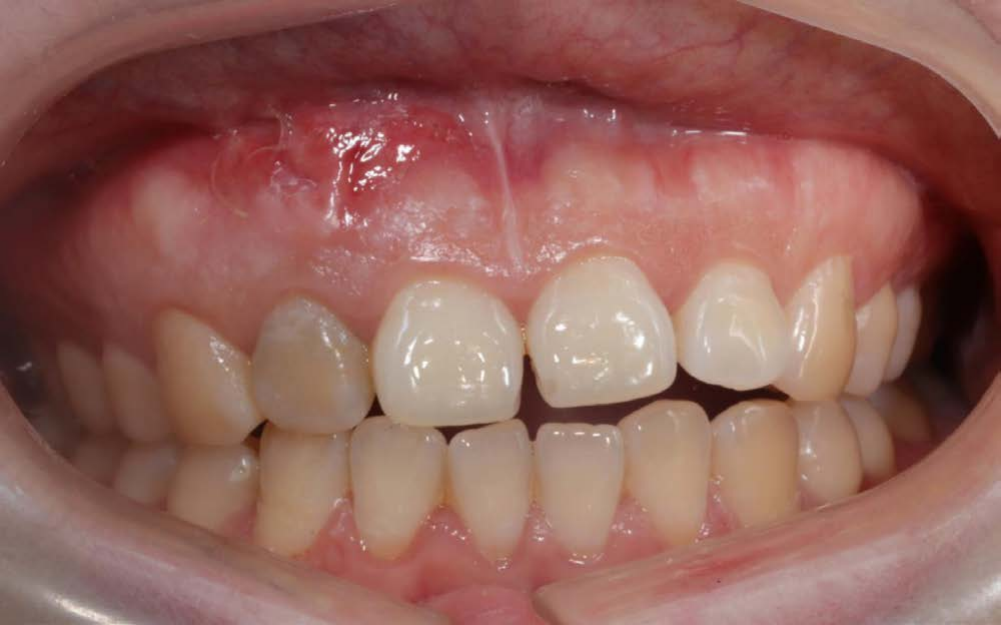
Uneventful healing without postoperative complications at 2-wk visit.

She received a normative RCT and calculus movement from the tooth apex (Fig. 3). The surgery approach was made through the mucosa fenestration and a sub-periosteum tunnel pouch about 10 mm × 20 mm around the fenestration was made with tunnel knives (Hu-Friedy). The tissue was released sufficiently so as to expose the bone defect area in the apex area. The exposed apex was scaled thoroughly using ultrasonic instruments and curettes (Gracey), and treated with ethylenediamine tetraacetic acid gel for 2 minutes. A diamond bur was used to prepare medullary foramina around the root apex (Fig. 4). A connective tissue graft (CTG) about 15 mm × 8 mm × 2.5 mm with entire periosteum was harvested from the palate with one–incision method.^[10]^ Then the CTG was pulled into the tunnel and stabled in the mesial side of the tunnel pouch with 5-0 sutures (Fig. 5). After that, a collagen bone graft (Geistlich) was trimmed and put into the bone defect. When the bone collagen was firmly stuffed, the other side of CTG was pulled into the tunnel pouch and stabled in the distal side of the tunnel. Finally, the fenestration approach was closed with a 5-0 polypropylene suture (Fig. 6). The majority of surgery was carried out under microscope and with microsurgery instruments. CBCT reconstruction was taken immediately after operation, which showed high-density particulate matter around the lower 1/2 part of the root (Fig. 7). Normative antibiotics were administrated for 5 days and the patient was advised rinsing 3 to 5 times per day with 0.12% chlorhexidine solution for 14-day postoperative maintenance. After 14 days, the patient was recalled and the sutures were removed. The healing was uneventful without postoperative complications (Fig. 8).

**Figure 9.**
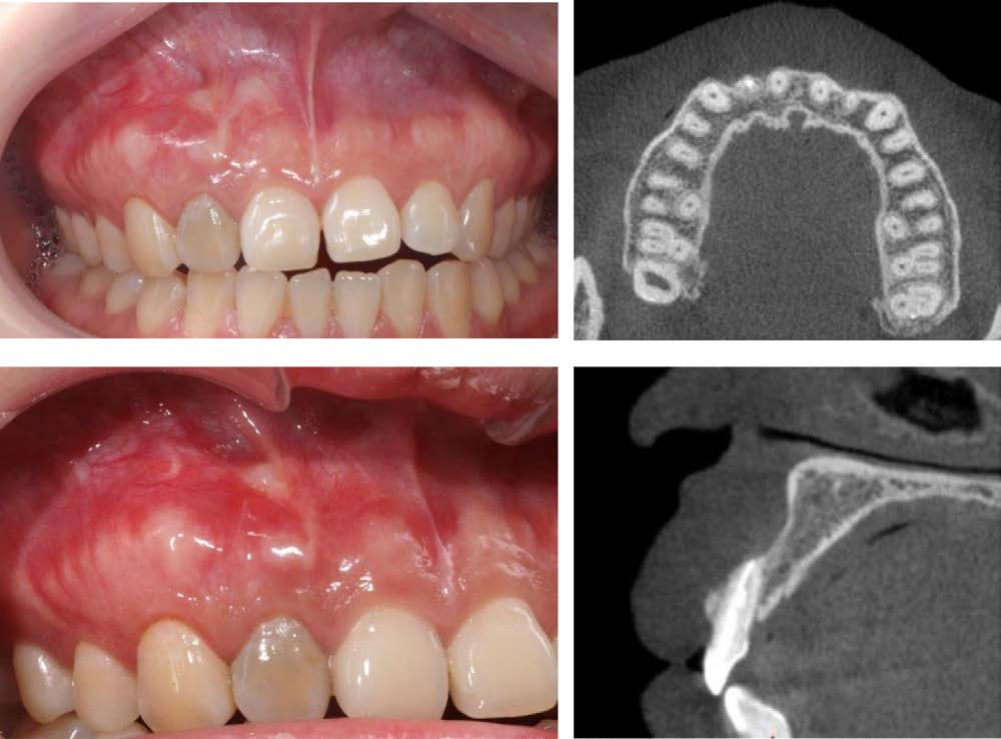
The intraoral photograph at 6-mo recall: thickened mucosa was obviously observed with a light color and touched stiffed. CBCT reconstruction showed that bone defect was restored at the apex with a continuous contour line. CBCT = cone-beam computed tomography.

**Figure 10.**
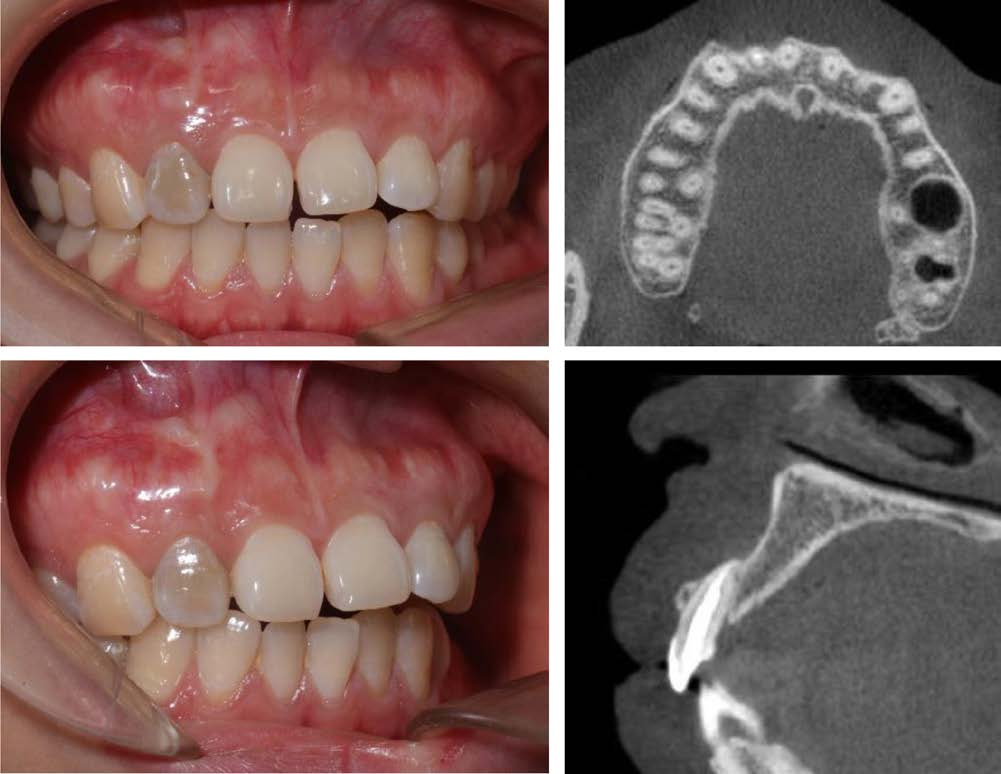
The intraoral photograph at 12-mo recalls: an acceptable aesthetic effect was gotten in the upper anterior teeth area. CBCT reconstruction showed stable bone restoration in the palatal aspect and the continuous lamina dura in the buccal aspect of #12 root apex. CBCT = cone-beam computed tomography.

At 6-month recall, the mucosa in the buccally apical region was healed and thicken obviously. CBCT showed the bone-like matter occupied the buccal aspect of #12 root apex with a continuous contour line (Fig. 9). At 12-month recall, an acceptable aesthetic effect was gotten in the upper anterior teeth area. CBCT showed that the continuous lamina dura occupied the buccal aspect of #12 root apex (Fig. 10).

## 3. Discussion

Mucosal fenestration concomitant with bone fenestration is also called gingivo-osseous pathologic fenestration, which refers to a window-like opening of the alveolar bone and the overlying mucosa. The majority of apical fenestrations occur on the buccal aspect of maxillary teeth.

Apical fenestrations may be caused by physiological factors. The bone defect in root apex region is a common anatomical variation, which may be caused by the thin alveolar bone or the tilted root. Apical fenestrations may also be caused by pathological processes, including endodontic disease, orthodontic treatment, periodontitis, tooth trauma, occlusal trauma.^[1]^ Base on the above mentioned, the most possible process in our case was that the buccally malpositioned root and thin alveolar bone caused the mucosal fenestration by persistent rubbing of the discomfort area and then the oral cavity communicated with the root canal system via the mucosal fenestration and apical foramina, which led to the biofilm formation on the exposed root and secondary pulp infection.

Compared with physiological reasons, apical fenestrations more often arise from primary endodontic infections and treatment failure, such as overfilling and over-instrumentation. And persistent periradicular inflammation could lead and aggravate the defect of the alveolar bone overlying the root apex. In this situation, root-end resection and retrofill are regarded as necessary and effective treatments to control the inflammation. Further, root resection could make the apex within the alveolar bone housing and improve visibility and accessibility for periapical curettage. In our case, according to the bone contour profile we assumed the etiology was the thin bone and mucosa phenotype rather than the primary endodontic reasons. Some occlusion factors as the open bite on #21-#23 were suggested to be considered but there was no certain idea. We eliminated the root apex resection upon good root canal treatment and well debridement to maintain the root length and mobility.

We realized that the etiology in this case might firstly be the physiological deficiency and then the pulp inflammation. So except for RCT and apex debridement removing the infection factors, the very important point was to accumulate the hard and soft tissue through a periodontal way, which was ignored in endodontic surgery, especially for the soft tissue. If mucosal fenestration is accompanied by alveolar bone loss and underlying pathology, mucogingival surgery of soft tissue grafting with bone regenerative therapy may achieve more desirable clinical results. A clinical study showed cases with CTG achieving complete soft tissue coverage while cases without CTG still having soft tissue openings postoperatively.^[11]^

It is reported that microsurgery design could improve the healing process and reduce post-surgery morbidity. The smallest surgery approach possible through fenestration and a limited tunnel pouch was performed to realize the minimally invasive concept of periodontal plastic surgery. So the intra-sulcular and vertical incisions were avoided to reduce the pain and scarring after operation. As this tunnel was made around the root apex, we named this technique “apical tunnel surgery” (Fig. 3).

Regeneration materials might be vital additive to the whole procedure to improve clinical outcomes. We assumed that it was difficult to ensure the stabilization of particle graft when the soft tissue graft was sliding into this limited surgical field. So a collagen bone graft instead of bone particles was chosen because of its better handling characteristics.

There are some options for the soft tissue management, including direct suturing, a pedicle flap and a free CTG. The soft tissue graft could release the excessive wound tension from large mucosal defect and GTR.^[4,11]^ Zucchelli reported a “soft tissue wall technique” in a case where the soft and hard tissue was designed to be accumulated in one surgery^17^. The CTG was placed upon the bone defect to stabilize the biomaterial enamel matrix derivative. It was encouraging but we are not sure whether the CTG could directly contact with the bone substitute material. In this situation, a biological membrane might be recommended to barrier the bone graft first. However, it would increase the difficulty in operation for the clinician and the cost for the patient. For this consideration, we performed a sub-epithelium method instead of de-epithelium way, so the CTG could be harvested with periosteum to barrier the soft-hard tissue interface. We paid great attention to harvest the graft with a 2 to 3 mm thickness to ensure the resistance to trauma and friction (Fig. 5). In fact, the thickened mucosa was obviously observed and felt sticky at the recall.

## 4. Conclusions

In conclusion, we report a successful clinical treatment of a root apex with both apical and mucosal fenestration. It indicates that apical fenestration caused by physiological deficiency could be treated with mucogingival surgery rather than endodontic surgery, with the aim to accumulate soft and hard tissue. We expect more cases and longer time follow-up to develop an evidence-based management procedure.

## Author contributions

**Conceptualization:** Chengjie Xie.

**Data curation:** Xiaohao Liu.

**Resources:** Chengjie Xie.

**Supervision:** Chengjie Xie.

**Validation:** Jingxin Weng.

**Writing – original draft:** Jie Chen.

**Writing – review & editing:** Gaoying Ran.
